# Antiparasitic Evaluation of Aquiluscidin, a Cathelicidin Obtained from *Crotalus aquilus*, and the Vcn-23 Derivative Peptide against *Babesia bovis*, *B. bigemina* and *B. ovata*

**DOI:** 10.3390/pathogens13060496

**Published:** 2024-06-10

**Authors:** Edwin Esaú Hernández-Arvizu, Masahito Asada, Shin-Ichiro Kawazu, Carlos Agustín Vega, Angelina Rodríguez-Torres, Rodrigo Morales-García, Aldo J. Pavón-Rocha, Gloria León-Ávila, Bruno Rivas-Santiago, Juan Mosqueda

**Affiliations:** 1 Immunology and Vaccine Research Laboratory, Natural Sciences College, Autonomous University of Queretaro, Queretaro 76230, Mexico; esau.hernandez@uaq.mx (E.E.H.-A.); jose.rodrigo.morales@uaq.mx (R.M.-G.); mvzaldo.pavon@gmail.com (A.J.P.-R.); 2 PhD Program in Natural Sciences, Natural Sciences College, Autonomous University of Queretaro, Queretaro 76230, Mexico; 3 National Research Center for Protozoan Diseases, Obihiro University of Agriculture and Veterinary Medcine, Inadacho, Nishi 2-13, Obihiro 080-8555, Hokkaido, Japan; masada@obihiro.ac.jp (M.A.); skawazu@obihiro.ac.jp (S.-I.K.); 4 Natural Sciences College, Autonomous University of Queretaro, Queretaro 76230, Mexico; carlos.vega@uaq.mx (C.A.V.); angelina@uaq.mx (A.R.-T.); 5 Department of Zoology, National School of Biological Sciences, National Polytechnic Institute, Carpio y Plan de Ayala S/N, C.P. 11340, Casco de Santo Tomas, Mexico City 11340, Mexico; leonavila60@yahoo.com.mx; 6 Medical Research Unit Zacatecas-Instituto Mexicano del Seguro Social, Zacatecas 98053, Mexico; rondo_vm@yahoo.com

**Keywords:** cathelicidin, antimicrobial peptide, babesia, in vitro culture

## Abstract

Babesiosis is a growing concern due to the increased prevalence of this infectious disease caused by *Babesia* protozoan parasites, affecting various animals and humans. With rising worries over medication side effects and emerging drug resistance, there is a notable shift towards researching babesiacidal agents. Antimicrobial peptides, specifically cathelicidins known for their broad-spectrum activity and immunomodulatory functions, have emerged as potential candidates. Aquiluscidin, a cathelicidin from *Crotalus aquilus*, and its derivative Vcn-23, have been of interest due to their previously observed antibacterial effects and non-hemolytic activity. This work aimed to characterize the effect of these peptides against three *Babesia* species. Results showed Aquiluscidin’s significant antimicrobial effects on *Babesia* species, reducing the *B*. *bigemina* growth rate and exhibiting IC_50_ values of 14.48 and 20.70 μM against *B*. *ovata* and *B*. *bovis*, respectively. However, its efficacy was impacted by serum presence in culture, and it showed no inhibition against a *B. bovis* strain grown in serum-supplemented medium. Conversely, Vcn-23 did not demonstrate babesiacidal activity. In conclusion, Aquiluscidin shows antibabesia activity in vitro and its efficacy is affected by the presence of serum in the culture medium. Nevertheless, this peptide represents a candidate for further investigation of its antiparasitic properties and provides insights into potential alternatives for the treatment of babesiosis.

## 1. Introduction

Babesiosis is a disease caused by intraerythrocytic parasites belonging to the genus *Babesia*. To date, over a hundred *Babesia* species have been identified, affecting both domestic and wild animals, with the species infecting livestock having the most significant impact [1,2] and, in this context, bovine babesiosis is the one with major economic consequences [3]. Recently, the number of cases of babesiosis in humans has increased, which has led it to be considered an emerging zoonosis [2]. *Babesia* parasites are transmitted by ticks [4]; however, in humans, one of the primary routes of infection is through blood transfusion [5], and there have been documented cases of vertical transmission in both humans and mice [6,7].

In many cases, infected patients remain asymptomatic [5], but certain factors predispose individuals to complications from the infection [8]. The multiplication of the parasite and the subsequent release of merozoites lead to erythrocyte lysis [8,9,10], resulting in symptoms primarily characterized by anemia, fever, hemoglobinuria, and fatigue [11,12]. Moreover, organ-related conditions can occur in patients infected with these intraerythrocytic parasites [13,14,15,16,17].

The most commonly used drugs for treating babesiosis in animal species are diminazene diaceturate and imidocarb dipropionate [9]. However, the use of diminazene is associated with evidence of prolonged elimination times and side effects like diarrhea [18], anaphylaxis, and nervous system signs [19]. Furthermore, some strains of *B*. *gibsoni* have also developed resistance to this drug [20,21]. Imidocarb treatment has been reported to cause pain and injection site reactions in a significant percentage of patients, along with gastrointestinal symptoms [19].

In the case of human babesiosis, treatments involve combinations of atovaquone/azithromycin or clindamycin/quinine. However, this treatment regimens have not consistently demonstrated effectiveness and patients with compromised immune systems may require supportive therapies, including blood transfusions [2]. Adverse effects have been reported for both therapy options, with a higher frequency observed in those receiving clindamycin/quinine [2,8]. All this is consistent with the need to develop and evaluate new compounds with anti-Babesia activity.

Antimicrobial peptides (AMPs), small molecules of the innate immune system, have been studied as potential antiprotozoal compounds due to their broad microbicide activity, and their role as immune response modulators [22], some of which have showed antiprotozoan activities against *Trypanosoma* spp., *Plasmodium* spp., and *Leishmania* spp. [23]. Cathelicidins, an important family of AMPs (antimicrobial peptides), mostly adopt an alpha-helical conformation upon contact with anionic phospholipids due to electrostatic attraction resulting from their cationic nature, which influences their microbicidal mechanism of action [24]. Aquiluscidin, a cathelicidin peptide, derived from the Queretaro dark rattlesnake, *Crotalus aquilus*, was identified by transcriptional analysis of oral mucosa epithelial cells and its amino acids sequence and physicochemical characteristics were predicted using bioinformatics tools. A derivative peptide, Vcn-23, was selected based on its predicted physicochemical properties for determining its antimicrobial activity. Both peptides were chemically synthesized and exhibited antibacterial effects in assays at concentrations <2 µM against several bacteria laboratory strains and human clinical isolates, demonstrating inhibitory effects against both Gram-positive and Gram-negative bacteria. Importantly, neither peptide showed hemolytic activity to erythrocytes even at concentrations up to 50 µM. Furthermore, Aquiluscidin and Vcn-23 were innocuous at low concentrations, maintaining 100% cell viability at concentrations of 12.5 µM [25]. Given these findings, and because they share properties with other cathelicidins that have been effective when evaluated against intracellular protozoa, both peptides are excellent candidates for further evaluation regarding their spectrum of activity and mechanisms of action. In this study, we assessed the in vitro anti-babesia activity of these two peptides against three different species that infect cattle, causing significant economic losses: *Babesia bigemina*, *Babesia bovis*, and *Babesia ovata*. Our findings identify Aquiluscidin as a promising molecule for the development of new therapeutic strategies.

## 2. Materials and Methods

### 2.1. Bioethics Approval

All protocols performed here and animal experimentation were approved by the Ethics Committee of the College of Natural Sciences at the Autonomous University of Queretaro, with the number 41FCN2020 on 1 July 2020. 

### 2.2. Source of B. bigemina and B. bovis Field Isolates

The *Babesia bigemina* isolate was obtained from infected ticks collected from cattle in the municipality of San Jose de las Torres, in the state of Michoacan, Mexico. The ticks were fed on a splenectomized steer and on 8 August 2017, the resulting infected blood was collected and stored in liquid nitrogen with DMSO (Sigma, St. Louis, MO, USA). The *Babesia bovis* isolate was obtained from infected ticks collected in the tropical bovine production unit “Santa Cruz”, in the municipality of Hueytamalco, in the state of Puebla, Mexico. The ticks were fed on a steer and, on 26 June 2019, the infected blood was also collected and stored in liquid nitrogen. Single species infection was confirmed by PCR in both cases. 

### 2.3. In Vitro Culture Adaptation

Splenectomy procedures were successfully conducted on two 10-month-old Holstein steers, each weighing 270 kg, at the Large Animal Veterinary Hospital of the Autonomous University of Queretaro, utilizing a specialized facility for ruminants. The unique methodological approach for splenectomy adapted by the hospital’s veterinarians included pre-surgical preparations such as fasting periods and procaine penicillin G (Shotapen^®^, Virbac, Mexico) administration [26]. Regional anesthesia techniques were used as reported by other authors [26,27,28]. Surgical techniques, including the incision process, layer penetrations, splenic hilum ligation, and closure methods, were carried out following previously described protocols [26,29,30]. Post-operative care involved pain management with Flunixin meglumine (Napzin^®^, PiSA Pharmaceuticals, Mexico), antibiotic therapy, wound monitoring, and dietary adjustments. Following the recovery period, uninfected blood was obtained from each animal using a sterile flask containing 10% (*v/v*) of glass beads. After a centrifugation step (1972× *g*, 15 min, 4 °C), serum was collected and stored at –80 °C until use. The cell package underwent washing, utilizing the same centrifugation protocol, with VyM solution [31] containing penicillin (10,000 units/mL), streptomycin (10,000 μg/mL), and fungizone (25 μg/mL) (Gibco, Invitrogen, Carlsbad, CA, USA). Washed erythrocytes were stored at 4 °C with VyM solution (1:1 *v/v*).

Once the cattle ended the post-operatory care, one of them was inoculated with 7 mL of blood infected with *Babesia bigemina* (Michoacan isolate), while the other was inoculated with 7 mL of blood infected with *Babesia bovis* (Puebla isolate); the animals were monitored for 8 and 15 days, respectively, until the desired level of parasitemia was reached, at which point blood was extracted.

Blood extraction occurred when parasitemia reached 1% (for *B*. *bigemina*) and 3% (*B*. *bovis*), using lithium heparin tubes. The blood underwent five washes with VyM solution. Subsequently, 100 µL of infected red blood cells were cultured with 1 mL of M199 (Sigma, St. Louis, MO, USA) supplemented with 40% bovine serum and 1% antibiotic-antimycotic in a 24-well plate at 37 °C and 4.7% CO_2_. Cell cultures were maintained under these conditions for 48 h. Following the initial culture, 800 µl of culture media was replaced daily, and 2 µL of the cell package was utilized to create smears for the assessment of infected cells. Every five days, the cultures were passaged and diluted at a 1:2 (*v/v*) ratio with new material, including healthy erythrocytes and fresh medium. This procedure was repeated iteratively until the parasites began to exhibit proper growth.

After the aforementioned procedures, normal erythrocytes from other 5 different non-splenectomized uninfected bovines were evaluated over a period of 2 weeks, analyzing the parasite growth rate during this time, to select the best candidate as a red cell donor for the maintenance of the in vitro cell culture. 

### 2.4. In Vitro Culture Maintenance 

Under strict sterile conditions, *Babesia bigemina* and *B. bovis* were maintained in vitro in the Immunology and Vaccines Laboratory at Autonomous University of Queretaro, as follows: 100 μL (10%, *v/v*) of washed infected red blood cells (iRBC) were cultured with 1000 μL of M199, supplemented with 40% bovine serum and antibiotic-antimycotic, in 24-well culture plates at 37 °C and 4.7% CO_2_; 800 μL of M199 was replaced daily, and subcultures were performed when the percentage of iRBC exceeded 3%.

For *B. ovata* (Miyake strain) [32] and a second strain of *B. bovis* (Texas strain) [33], in vitro cultures were maintained at the National Research Center of Protozoan Diseases in Obihiro, Japan. They were cultured in GIT medium (without serum) with 10% (*v/v*) iRBC in 24-well plates and incubated at 37 °C with 5% O_2_ and 5% CO_2_. Culture media were replaced daily, and passages were carried out when the percentage of iRBC reached 6%. 

In all cases, the parasite growth rate was evaluated to determine the optimal initial infected red blood cell percentage (iRBC%) for the antimicrobial assays.

### 2.5. Antiparasitic Activity Assay

Aquiluscidin (KRFKKFFKKVKKSVKKRLKKIFKKPMVIGVSFPF-amidated, 4171.32 Da, net charge +16) and Vcn-23 (FFKKVKKSVKKRLKKIFKKPMVI-amidated, 2848.72 Da, net charge +12). The synthetic peptides were acquired from Peptide 2.0, with procedures and characteristics as previously reported [25]. In preparation for subsequent utilization, they were reconstituted in sterile water, reaching a final concentration of 2 mg/mL.

The antiparasitic activity was assayed against intraerythricytic Babesia’s stages (merozoites and trophozoites) of four species. *B*. *bigemina* (Michoacan isolate) and B. *bovis* (Puebla isolate) were both supplemented with bovine serum as follows: In a 96-well plate, 20 μL of 1% iRBC were cultured in each well. Lyophilized peptides were dissolved in sterile ultra-pure water and stored under refrigeration. A total of 200 μL of M199 with 40% bovine serum containing different concentrations of Aquiluscidin and Vcn-23 (1.25–25 μM) or imidocarb dipropionate (2 μM) was added. Two negative controls consisted of iRBC cultured with M199 with 40% bovine serum alone and with the highest volume of sterile water corresponding similarly to the amount of added peptide at its maximum concentration. Culture media were replaced every 24 h for each treatment, and smears were made from each well, fixed with absolute methanol and stained with Giemsa for 20 min. More than 2000 cells were counted in each stained smear, and the percentage of iRBC was calculated. The procedure was repeated for four consecutive days, with all treatments performed in triplicate and in two different experiments.

For *B. bovis* (Texas strain) and *B. ovata* (Miyake strain), non-serum cultured parasites were maintained in 24-well plates. For the antimicrobial assay, a 96-well cell culture plate was used. Aquiluscidin and Vcn-23 were applied at different concentrations (1.56–25 μM). Diminazene aceturate (DA) (Sigma-Aldrich, Tokyo, Japan) was dissolved in DMSO (Fujifilm Wako, Chuo-Ku, Osaka, Japan). Treatments were prepared in triplicate, and infected erythrocytes were adjusted to 0.2%. An amount of 20 µL of adjusted iRBC was cultured with 200 µL of GIT medium mixed with the peptides and DA at specified concentrations. Three negative controls were used: GIT medium alone, GIT medium with ultrapure water at the same volume as the highest peptide concentration, and GIT medium with DMSO at the same volume used for DA. Cells were incubated for 24 h under the same conditions described previously. Subsequently, 200 µL of medium containing the evaluated components (peptides, DA, and controls) was replaced daily, and smears of the cell package (1–2 µL) were made to count the infected erythrocytes. The smears were fixed with methanol and stained with Giemsa (Merk KGAa, Darmstadt, Germany). All experiments were conducted in triplicate and had a total duration of 96 h. To determine the percentage of infected erythrocytes, at least 2000 cells were counted using a light microscope. 

### 2.6. Statistical Analysis

Data were analyzed using descriptive statistics and one-way ANOVA. IC_50_ was calculated by non-linear regression using GraphPad Prism 8 software.

## 3. Results

### 3.1. Selection of Healthy Bovine Erythrocyte Donors 

For the maintenance of the *B*. *bigemina* culture (Michoacan isolate), uninfected erythrocytes from five donor cattle were evaluated to select the best candidate. As shown in Figure S1, after 96 h of growth, the serum from the individual identified with the number 9180 induced the highest amount of parasitized red cells in the in vitro culture with 2.98% (±0.1002). Statistical analysis (ANOVA with Tukey’s post hoc test) showed a significant difference with the results obtained for the rest of the evaluated animals, except for number 8183. Bovine 9180 was selected as the donor of erythrocytes for the *B*. *bigemina* culture.

In the case of *B*. *bovis* (Puebla isolate), it was not possible to carry out the selection, so the parasites were maintained using the serum and erythrocytes of the same donor animal (collected before experimental infection) for the total duration of the experiment. 

### 3.2. Antiparasitic Effect

**Figure 1:**
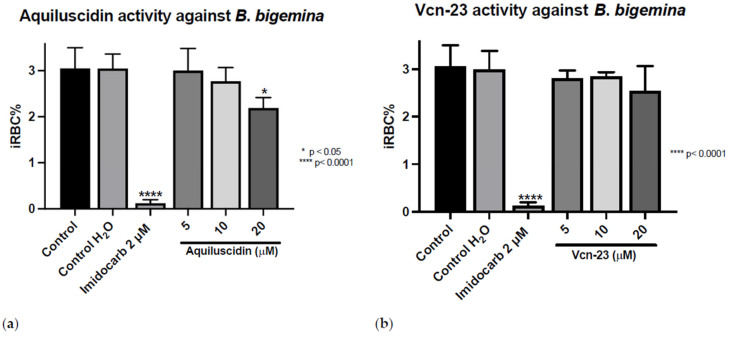
Antiparasitic activity of Aquiluscidin and Vcn-23 against *B. bigemina* (Michoacan strain) in vitro. (**a**) Activity of Aquiluscidin and (**b**) activity of Vcn-23 against *B*. *bigemina* after 96 h. The assay was conducted in triplicate, and the data are presented as the mean and standard deviation. iRBC%: percentage of infected red blood cells. Three control groups were included: control: culture to which no additional treatment or substance was added; control + H_2_O: water (the diluent for the peptides), was added to the cultures with a volume equivalent to the highest evaluated concentration; imidocarb: 2 μM. * *p* < 0.05, **** *p* < 0.0001.

The antiprotozoal activity was tested in vitro against three different *Babesia* species (Figure S2). The treatments were applied and the iRBC% was calculated after 96 h. As it is shown in Figure 1, in the control group that did not receive any treatment, the average parasitic growth rate was 3.053% (±0.445); in the control group to which water was added in the same volume as the highest peptide concentration used, this growth rate was 3.05% (±0.3122). For the control group treated with imidocarb at 2 µM, the mean growth rate was 0.1233% (±0.07638). For Aquiluscidin, at concentrations of 5 and 10 µM, parasitic growth rates of 3.00% (±0.4828) and 2.773% (±0.2954) were obtained, respectively. Neither of these treatments resulted in statistically significant efficiency after ANOVA with Dunnett’s test (*p* < 0.05). Aquiluscidin had activity against *B*. *bigemina* in the in vitro inhibition assay, at a 20 µM concentration, with a percentage of 2.19 erythrocytic parasites (±0.2254). This corresponds to a decrease of 28.19% of iRBC compared with the controls (Figure 1a). The results obtained with the addition of Vcn-23 at concentrations of 5, 10, and 20 µM showed parasitic values of 2.803% (±0.1650), 2.840% (±0.09644), and 2.537% (±0.5292), respectively (Figure 1b). Although there was a slight decrease in the number of parasites identified during the count, none of the treatments showed a statistically significant difference compared to the control. 

**Figure 2:**
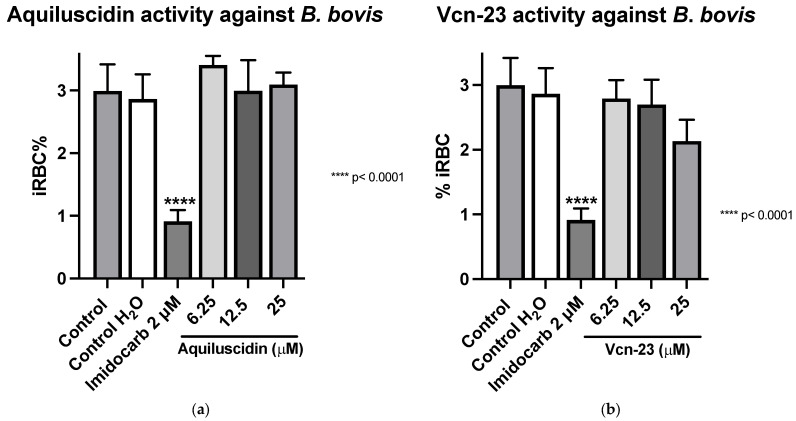
Antiparasitic activity of Aquiluscidin and Vcn-23 against *B. bovis* (Puebla isolate) in vitro. (**a**) Activity of Aquiluscidin and (**b**) activity of Vcn-23 against *B*. *bovis* after 96 h. The assay was conducted in triplicate, and the data are presented as the mean and standard deviation. iRBC%: percentage of infected red blood cells. Three control groups were included: control: culture to which no additional treatment or substance was added; control + H_2_O: water (the diluent for the peptides), was added to the cultures with a volume equivalent to the highest evaluated concentration; imidocarb: 2 μM. **** *p* < 0.0001.

Regarding the evaluation of antimicrobial activity against *B*. *bovis* (Puebla isolate) (cultured with M199 + 40% bovine serum), the results obtained showed that in the case of Aquiluscidin (Figure 2a), it is evident that none of the evaluated concentrations exhibited inhibitory activity when compared to the untreated growth controls. Regarding the activity depicted in Figure 2b, by the Vcn-23 peptide, there is a slight decrease in the percentage of infected erythrocytes with peptide treatments at different concentrations compared to the growth controls. This reduction becomes more pronounced at the highest concentration evaluated (25 μM); however, none of the treatments showed statistically significant differences with the control groups. These results indicate that neither Aquiluscidin nor Vcn-23 exert in vitro inhibitory activity in the growth of this *B*. *bovis* isolate. 

**Figure 3:**
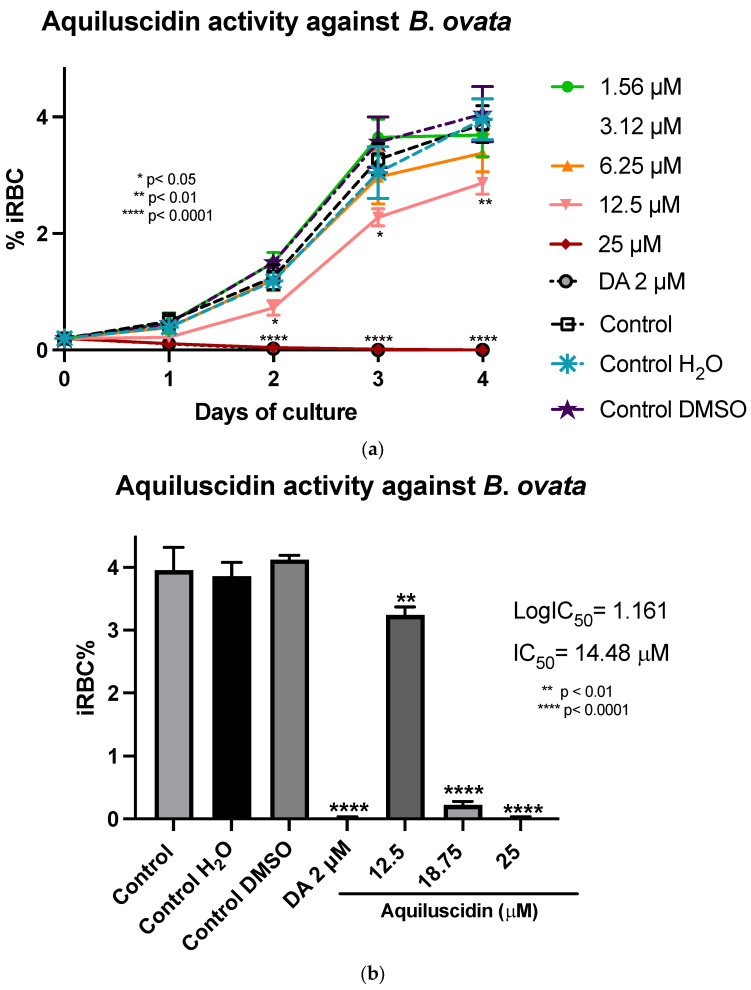

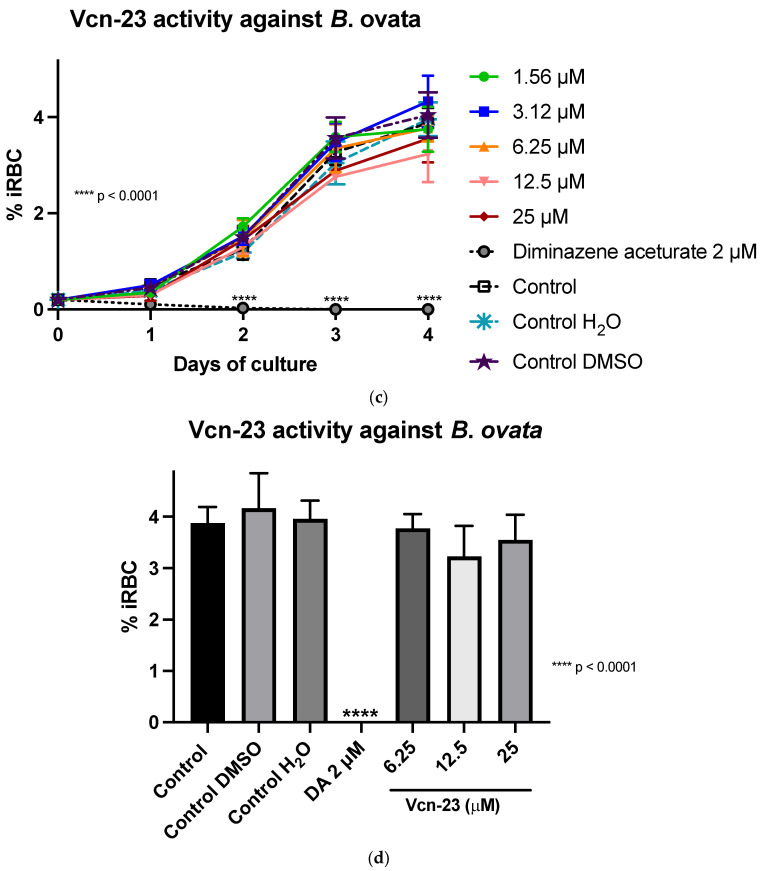
Antiparasitic activity of Aquiluscidin and Vcn-23 against *B. ovata* (Miyake strain) in vitro. (**a**) Activity of Aquiluscidin (1.56–25 µM) and diminazene aceturate (DA) (2 µM) over the course of four days. (**b**) Calculation of Aquiluscidin IC_50_ using three concentrations where inhibition was observed, determined through nonlinear regression at 96 h. (**c**) Activity of Vcn-23 and DA over the four days. (**d**) Activity of Vcn-23 and DA at 96 h, only highest concentration testes are plotted. The percentages for each treatment represent the mean of triplicate measurements, with error bars indicating the corresponding standard deviations. iRBC%: percentage of infected red blood cells. DA: diminazene aceturate. Three control groups were included: control: culture to which no additional treatment or substance was added; control + H_2_O: water (the diluent for the peptides), was added to the cultures with a volume equivalent to the highest evaluated concentration; control + DMSO: the diluent for DA, using the same volume as the drug treatment. * *p* < 0.05, ** *p* < 0.01, **** *p* < 0.0001.

In the assays conducted with *B*. *ovata* (Miyake strain) using a serum-free medium (GIT), Aquiluscidin exhibited inhibitory growth effective at 12.5 and 25 µM during all the experimental days (Figure 3a). The antibabesia activity of Aquiluscidin was notorious and at 25 µM, parasites were completely cleared from the second day in culture. At 12.5 µM, Aquiluscidin reduced parasite growth by 38.75%, 25.27%, and 27.60% on days 2, 3, and 4, in culture, respectively. To calculate the Aquiluscidin IC_50_ for the parasite growth, a second inhibitory assay was performed using 12.5, 18.75, and 25 µM under the same conditions of the previous experiment. The iRBC obtained at 96 h were counted and the IC_50_ was calculated by non-linear regression. Aquiluscidin decreased *B*. *ovata* growth by 16.91% and 94.22%, at 12.5 and 18.75 µM, respectively (Figure 3b); and at 25 µM it eliminated the parasites, with an IC_50_ of 14.48 µM. The result for Vcn-23 was totally different, as this peptide was not able to inhibit the parasite growth (Figure 3c,d). This outcome did not change even after increasing the concentration up to 50 µM. 

**Figure 4:**
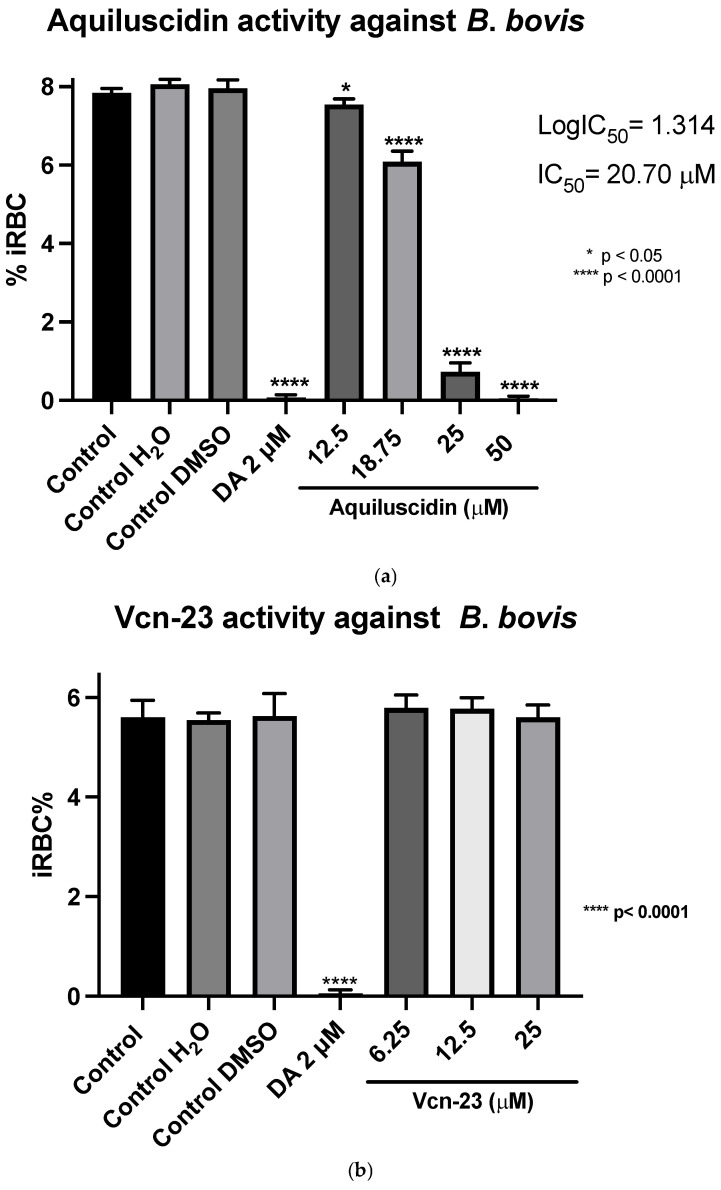
Antiparasitic activity of Aquiluscidin and Vcn-23 against *B*. *bovis* (Texas strain) in vitro. (**a**) Determination for Aquiluscidin IC_50_ using four concentrations where inhibition was observed, determined through nonlinear regression at 96 h. (**b**) Activity of Vcn-23 and diminazene aceturate (DA) after 96 h. The percentages for each treatment represent the mean of triplicate measurements, with error bars indicating the corresponding standard deviations. iRBC%: percentage of infected red blood cells. DA: diminazene aceturate. Three control groups were included: control: culture to which no additional treatment or substance was added; control + H_2_O: water (the diluent for the peptides), was added to the cultures with a volume equivalent to the highest evaluated concentration; control + DMSO: the diluent for DA, using the same volume as the drug treatment. * *p* < 0.05, **** *p* < 0.0001.

Regarding the second strain of *Babesia bovis* (Texas strain), cultured at the same conditions as *B*. *ovata*, using GIT medium in absence of bovine serum, the two highest concentrations had an antiparasitic effect, in a similar way to *B*. *ovata*. Low concentrations of Aquiluscidin were unable to inhibit the parasite growth. Considering these results, another experiment to calculate the IC_50_ was designed; four Aquiluscidin concentrations were used and cells of smears from the last evaluated day were counted. *B*. *bovis* growth decreased by 6.45%, 24.54%, 90.99%, and 100% using the peptide concentrations of 12.5, 18.75, 25, and 50 µM, respectively, when compared with the controls (Figure 4a). The calculated IC_50_ was 20.74 µM. Regarding Vcn-23 activity, it is important to mention that during the initial days of the assay, a decrease in the number of parasites observed under the microscope was noticeable at 25 µM. This activity was not sustained throughout the duration of the experiment and no detectable inhibitory effect was observed at any of the concentrations evaluated at 96 h (Figure 4b). 

Diminazene aceturate eliminated both *B*. *bovis* and *B*. *ovata* at 2 µM, while DMSO and water did not interfere with parasite normal growth. Table 1 shows a comparative analysis of IC_50_ obtained for Aquiluscidin and other compounds studied against *Babesia* spp.

## 4. Discussion

*Babesia* is an intraerythrocytic parasite that affects humans and animals, causing significant economic losses in livestock species [1,2]. Current veterinary treatments, diminazene aceturate and imidocarb, have several side effects and long withdrawal times; moreover, they are not always effective at eliminating the parasites. Furthermore, the use of drugs to control vector-borne diseases, which is the main strategy in the fight against protozoan infections, is also the cause of the generation of resistance, increasing the magnitude of the problem. [9,18,19,20,23]. This situation has motivated the research and development of new babesiacidal components and various compounds have been evaluated for this purpose [39]. Antimicrobial peptides (AMP’s) have been identified from different sources, most of them come from animal species [22]. Some of these molecules have been evaluated against protozoan parasites [23], including *Plasmodium* spp. a similar model to *Babesia* [40]. Aquiluscidin is a cathelicidin peptide identified in *Crotalus aquilus* from skin and oral mucosa biopsies; Vcn-23 is a shorter derivative peptide designed by considering its physicochemical properties. Both molecules showed a good antibacterial activity and non-hemolytic effects with a similar performance in those assays [25]. In this work, we tested these peptides against the main *Babesia* species infecting cattle, with a significant economic impact on the livestock sector: *B. bovis*, which is highly pathogenic and has a global distribution; *B*. *bigemina*, one of the primary causative agents of bovine babesiosis with the highest prevalence worldwide, due to its rapid dispersion and longer duration in the bovine bloodstream, leading to hemolytic anemia, and *B*. *ovata* which is a parasite distributed in East Asia, causing infections in immunocompromised animals, and prompting research for improved control strategies [41,42,43].

Aquiluscidin presented antibabesia activity in a dose-dependent manner against *Babesia bovis* and *Babesia ovata* with an IC_50_ of 20.74 and 14.48 µM, respectively, and complete inhibition of parasitic development achieved at the highest concentration used against each species. Both parasites were cultured with GIT, a serum free medium. Regarding the field isolates, *B*. *bigemina* and *B*. *bovis*, cell culture was supplemented with 40% bovine serum, and Aquiluscidin was only effective against *B*. *bigemina* at the highest concentration tested (20 µM). In the case of this *B*. *bovis* isolate, this cathelicidin did not have any inhibitory activity. 

Our findings highlight that the amount of serum used in in vitro assays significantly influences the parasiticidal function of Aquiluscidin. This aspect represents one of the primary challenges when considering peptide-based therapy. Some authors mention that many antimicrobial peptides can be affected by the presence of proteases in the serum, which reduces their effectiveness in carrying out their microbicidal action. This trouble can also be attributed to high-salinity conditions caused by serum components [44,45,46]. 

Additional components in serum, such as lipoproteins or albumin, may impede the efficacy of antimicrobial peptides [47]. Furthermore, the influence of peptides like defensins has been examined under varying serum concentrations in cell culture medium, revealing a 30% or 75% decrease in activity with 1% or 5% bovine fetal serum, respectively [48].

One of the solutions explored to address this issue is the chemical modification of peptide structures, aiming to enhance their antimicrobial activity, stability, and resistance while minimizing impact on healthy host cells [22,49]. Both Aquiluscidin and Vcn-23 were synthesized with a C-terminal amidated residue [25]. This modification in α-helical peptides is associated with increased antimicrobial potential, secondary structure formation, resistance to degradation, and membrane-crossing due to the increase in electrostatic attraction [22,50,51,52,53]. In other peptides, this alteration has enhanced their parasiticidal action against protozoa like *L*. *major* [54].

Despite the modification of Aquiluscidin, a decrease in its microbicidal potential in the presence of serum could not be avoided. It has previously been demonstrated that in linear peptides, C-terminal amidation alone is insufficient to evade the action of serum carboxypeptidases [49]. This reduction in microbicidal activity may also result from interactions with serum components, which has been previously reported for other types of AMPs (antimicrobial peptides). Aquiluscidin and Vcn-23 share structural characteristics (N-terminal helical arrangement followed by a hydrophobic fragment, in addition to the C-terminal amidation), which in other snake cathelicidins, facilitate interactions with serum components, such as albumins, apolipoproteins, and globulins [53,55]. Both factors contribute to an increase in the effective concentrations of AMPs when their effects are evaluated in the presence of high serum levels [49]. 

Cathelicidins and its derivative peptides have shown activity against different protozoan parasites, like *Trypanosoma* spp. and *Plasmodium* spp., with IC_50_ values in the range of 3.045–33.1 µM. Many of these assays were conducted with serum percentages ≤10% in cell culture, allowing for better direct antimicrobial activity [40,56,57,58]. This phenomenon has also been observed in the treatment with other antimicrobial proteins [59]. Moreover, it has become evident that the effectiveness of AMPs varies depending on the parasite’s developmental stage [60], and at the same time, a higher peptide concentration is required to eliminate intracellular stages. For example, several AMPs have been shown to eradicate *Leishmania* spp. promastigotes; however, similar concentrations do not exhibit the same efficiency against amastigotes, highlighting the greater challenge in targeting the intracellular phases of protozoa [61]. Additionally, IC_50_ also increases with higher serum usage in cell culture [57]. It is noteworthy that different experimental conditions, peptide types, and species determine the antimicrobial effectiveness of cathelicidins. 

Despite this, it is possible to explain why Aquiluscidin did show antimicrobial activity against *B*. *bigemina* at the highest concentration tested. Crotalicidin, a cathelicidin peptide from South American rattle snake, had 71 min of serum half-life, and its smaller derived molecule had at least 21 min [62]. Moreover, cathelicidin’s antiparasitic assays with other protozoan models (*Trypanosoma* spp.) caused affectations just after 15 min of treatment [56]; this means that antimicrobial peptides only need a few minutes after contact with the pathogen to have a microbicidal effect; so, the time Aquiluscidin remains active in the serum would be enough to carry out its activity.

Conversely, none of the peptides were effective in inhibiting the growth of a virulent, field isolate of *B. bovis*. In addition, it is important to consider factors related to the pathogen against which antimicrobial activity is being evaluated; in this case, *B. bovis*. A derived peptide of longicin, a tick AMP, was able to inhibit *B*. *bigemina* growth, but it was not effective against *B*. *bovis*. This result was attributed to the different experimental conditions for the cultivation of both species, as well as the differences in the size of the two parasites, which would affect the number of merozoites obtained after each binary fission process, favoring the greater development of *B*. *bovis* in this case [63]. The greater resistance of *B*. *bovis* to the susceptibility of AMPs may also be linked to its higher degree of pathogenicity, compared with other *Babesia* species [64,65]. In other protozoan models, metalloproteases have been identified as virulence factors that protect them from various host immune mechanisms, including the activity of cathelicidins and defensins, thus promoting their survival [66]. *B. bovis* is also known to produce enzymatically active metalloproteases, the inhibition of which affects its ability to grow [67]. Furthermore, it has been described that the susceptibility of *B*. *bovis* to components with antibabesial activity depends on the amount of serum used to supplement the in vitro culture. Therefore, a lower serum percentage in the medium requires a lower drug concentration to inhibit the growth of this parasite [33]. This could be related to the lower inhibitory effect of imidocarb dipropionate (2 µM) on *B*. *bovis* compared to *B*. *bigemina*. These factors are likely key contributors to its heightened resistance to the microbicidal effects of peptides.

It has been demonstrated that erythrocytes undergo a series of changes in their membrane structure once they have been infected with intracellular parasites. Among these changes, a serrated appearance has been observed, with a surface consisting of protrusions and indentations. These modifications could be related to the adhesion of erythrocytes to vascular endothelium [68,69]. Alterations in the shape of the erythrocyte plasma membrane may vary depending on the species that infects them, but in general, they include fissures, invaginations, vesicle formation, and protrusions. A probable function of these modifications is the transport and expression of parasite antigens and proteins (which may be necessary for adhesion to endothelium) on the surface of red blood cells [70]. Phospholipids in the membranes of erythrocytes infected with *P*. *falciparum* undergo modification in terms of the type and quantity compared to uninfected red cells. This would result in an increase in membrane fluidity and a configuration that approximates that of trophozoites and schizonts [71]. Some of these changes are similar to the ones observed in bovine red blood cells infected with *Babesia bovis* [72]. Collectively, these conformational changes would be fundamental for antimicrobial peptides to have a selective effect on infected erythrocytes and, additionally, to show affinity for the parasite’s membrane within the erythrocyte [59,73].

Vcn-23 did not significantly inhibit the growth of any species. Despite reducing the counted parasites in one of the *B*. *bovis* assays, where Aquiluscidin had no effect, it could not be considered effective in the statistical analysis. Previously, this molecule had exhibited potent antibacterial activity, in some cases better than the peptide from which it was derived, while showing lower cytotoxic and hemolytic potential [25]. The lack of activity in comparison to Aquiluscidin can be attributed primarily to its reduced cationic character, a crucial feature in antimicrobial peptides. Decreases in activity have been observed in several AMP derivatives that, due to modifications in size, sequence, and physicochemical properties compared to their parent peptides, tend to be less efficient or fail to inhibit pathogen growth [74]. Similar situations arise when evaluating peptide fragments, where it becomes evident that higher concentrations are required for inhibiting microorganism growth compared to the original peptide [62]. This has also been reported when evaluating the activity against protozoa; they have even been 150-fold less effective than native AMPs [57,75]. Moreover, Vcn-23 also has the previously described properties that allow it to interact with serum components.

The IC_50_ of Aquiluscidin against the *Babesia* species was effective compared with those values obtained with other compounds studied for the same purpose, whose range falls between 4.7 and 206 µM (Table 1); this indicates that Aquiluscidin has a good babesiacidal effect. This peptide was innocuous up to 50 µM against mammals’ erythrocytes; meanwhile, cytotoxic assay with HEK293 cells revealed a LD_50_ of 26.31 µM [25], which is close to the IC_50_ attained in this work. Additionally, its direct antimicrobial potential is mainly demonstrated in serum-free assays, making it challenging to evaluate them under physiological conditions. 

The fact that the capacity of AMPs to eliminate microorganisms is affected under physiological or pathological conditions (such as high salt concentrations and excessive mucus production) has been postulated that this may not be their only biological function. In in vivo assessments, some peptides have not displayed direct antimicrobial activity, yet they have provided protection through other mechanisms of immune regulation, leading to them being referred to as host defense peptides (HDPs) [76,77]. Regarding the aspect of immunomodulation, an advantage has been described in that these peptides do not directly interact with cell membranes, resulting in lower rates of hemolysis and cytotoxicity; moreover, they require lower doses than that for the direct antimicrobial activity and it helps to avoid the appearance of resistant microorganisms, because their interactions would be with the immune receptors and not directly with microbes. This function can be exerted under the conditions mentioned above [77,78,79,80,81]. Therefore, there is an emphasis on understanding the structure of these molecules and designing derivatives with better activity [45]. Cathelicidins are among the most extensively studied peptides to identify their immunomodulatory potential related to both innate and adaptive immune responses [79,80,82]. Shorter derived molecules, most of them without a microbicidal effect, called innate defense regulators (IDR), have been developed and have shown modulation of immune responses and protective activities against distinct pathogens, including protozoan parasites [77,83,84]. This characteristic is one of the most important advantages that peptide-based therapy has over conventional antimicrobial agents. Despite the fact that we did not test this, Aquiluscidin and Vcn-23 still could exert modulator activities, which could improve their potential as antibabesial molecules. 

In conclusion, we identified the direct antiprotozan effect of Aquiluscidin, the cathelicidin peptide from *Crotalus aquilus*, against *Babesia* spp., in a dose-dependent manner. Although its inhibitory effect is altered by the presence of serum in cell culture, this molecule represents a potential candidate for new drug development against babesiosis. 

## Figures and Tables

**Table 1:** Comparative analysis of IC_50_ obtained for Aquiluscidin and other compounds studied against *Babesia* spp.

Antibabesia Compound	IC_50_ (µM) against *B*. *bigemina*	IC_50_ (µM) against *B*. *bovis*	IC_50_ (µM) against *B*. *ovata*	Reference
*Syzygium aromaticum* (clove)	8.7	109.8	-	Batiha et al., 2019 [34]
*Camellia sinensis* (green tea)	71.3	114	-	Batiha et al., 2019 [34]
Nerodilol	29.6	21	29.6	AbouLaila et al., 2010 [35]
Ciprofloxacin derivative:				Batiha et al., 2020 [36]
3	13.7	32.9	-	
5	25.8	14.9	-	
10	33.9	34.9	-	
14	28.3	26.7	-	
15	26.6	4.7	-	
Atranorin	64.5	98.4	-	Beshbishy et al., 2020 [37]
Clindamycin	206	126.6	-	AbouLaila et al., 2012 [38]
Aquiluscidin	=	20.70	14.48	This study

-: Data not available; =: IC_50_ value was not obtained.

## Data Availability

Data are contained within the article.

## Supplementary Materials

The following supporting information can be downloaded at https://www.mdpi.com/article/10.3390/pathogens13060496/s1, Figure S1. Analysis of *B*. *bigemina* growth with erythrocytes from 5 donor bovines.; Figure S2. Representative Giemsa-stained smears of intraerythrocytic stages of *Babesia* species utilized in antiparasitic assay.
