# Supplementary material for: Antiparasitic Evaluation of Aquiluscidin, a Cathelicidin Obtained from Crotalus aquilus, and the Vcn-23 Derivative Peptide against Babesia bovis, B. bigemina and B. ovata
Source: Pathogens. 2024 Jun 10;13(6):496. doi: 10.3390/pathogens13060496 (PMC11206629; doi:10.3390/pathogens13060496)
Supplement: Supplementary file 1 [file pathogens-13-00496-s001.zip › pathogens-2957953-supplementary.pdf]

Supplementary Material

Evaluation of *B. bigemina* growth after 96 h

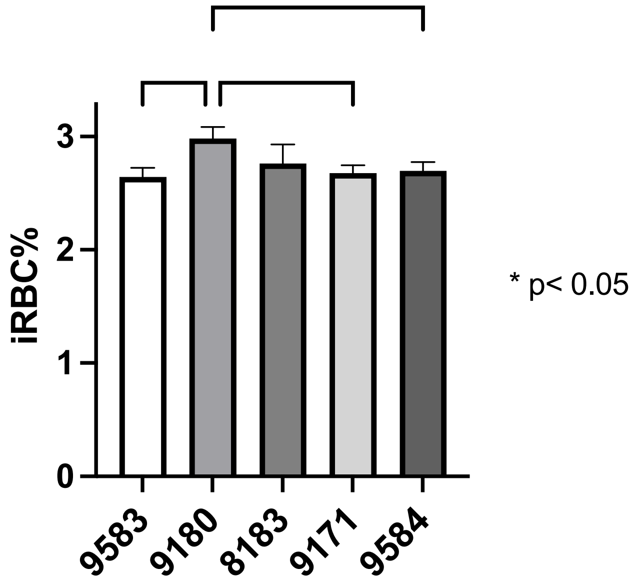

Figure S1. Analysis of *B. bigemina* growth with erythrocytes from 5 donor bovines. The X-axis shows the identification numbers of the evaluated individuals. After 96 h, the percentage of infected erythrocytes was determined. An ANOVA with Tukey's post hoc test was used for statistical analysis. iRBC%: Percentage of infected red blood cells. *$p < 0.05$

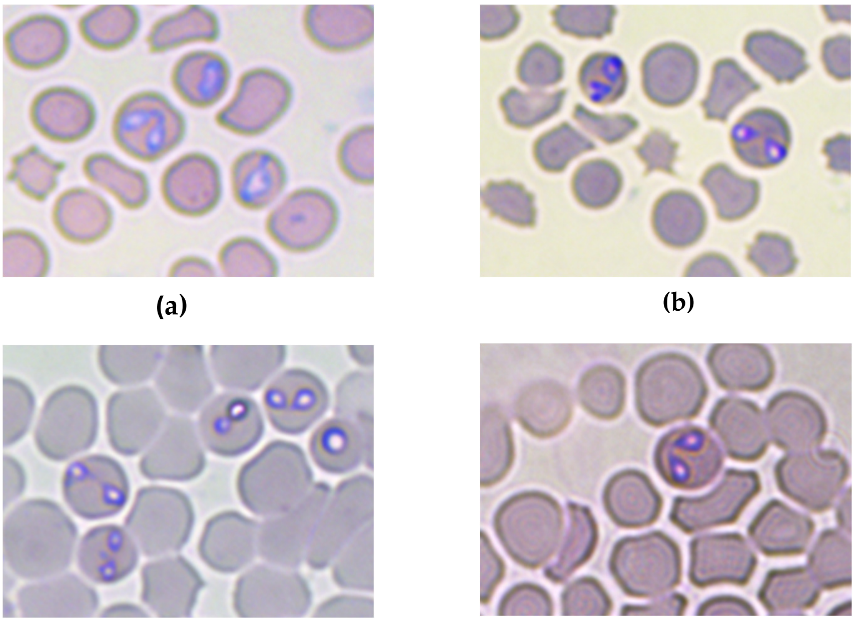

Figure S2. Representative Giemsa-stained smears of intraerythrocytic stages of *Babesia* species utilized in antiparasitic assay. (a) *B. bigemina* (Michoacan strain). (b) *B. bovis* (Puebla strain). (c) *B. bovis* (Texas strain). (d) *B. ovata* (Miyake strain).
